# Characteristics and PD-1 expression of peripheral CD4^+^CD127^lo^CD25^hi^FoxP3^+ ^Treg cells in chronic HCV infected-patients

**DOI:** 10.1186/1743-422X-8-279

**Published:** 2011-06-07

**Authors:** Tao Shen, Jiajia Zheng, Hua Liang, Chunhui Xu, Xiangmei Chen, Ting Zhang, Qiang Xu, Fengmin Lu

**Affiliations:** 1 Department of Microbiology, Peking University Health Science Center, Beijing 100191, China; 2 State Key Laboratory for Infectious Disease Prevention and Control, National Center for AIDS/STD Control and Prevention, Chinese Center for Disease Control and Prevention, Beijing 102206, China

## Abstract

**Background:**

Both regulatory T cells (Tregs) and PD-1/PD-L1 pathway were critically involved in HCV viral persistence. However, the association between them was not well investigated. Herein, we aimed to investigate the distributional profiles of Tregs subsets and association between PD-1 expression on these subsets and development of HCV long-term persistence.

**Methods:**

CD45RA and CD27 were employed to separate peripheral Tregs as naïve/central memory/effector memory/effector subsets. The phenotypic characteristics and PD-1 expression of Tregs were studied by flow cytometry.

**Results:**

In the present study, the majority of Tregs was identified as central memory phenotype in chronic hepatitis C patients compared with nearly equal contribution of naïve and central memory subsets in healthy individuals. PD-1 expression was elevated in all CD4+ T cell subset in chronic HCV infected patients, including Tregs. Of note, higher level of PD-1 expression was found on TEM- and effector-Treg than naïve- and TCM-Tregs subsets. The ratio of TEM-Tregs/naive-Tregs and TEM-Tregs/TCM-Tregs regarding to PD-1 MFI were significantly lower in CHC patients compared to controls.

**Conclusions:**

Our study indicated that distinctive characteristics of PD-1 expression on Tregs in HCV infection suggests associated with impaired adaptive immunity as well as viral long-term persistence. The cross talk between Treg cells and PD-1 induced inhibition in chronic HCV infection deserved further exploration for HCV infection associated immune pathogenesis.

## Introduction

Hepatitis C virus (HCV) infection presents a major global health challenge, with an estimation of more than 180 million infected patients worldwide and three to four million newly-infected cases annually [1,2]. Although some breakthroughs were reached recently in the treatment of hepatitis C [3-5], HCV infection is still far away from effective prevention by vaccination and has become one of the most important causes of chronic hepatitis, cirrhosis, and hepatocellular carcinoma around the world.

It is well documented [6-8]that duration and intensity of HCV-specific CD4^+ ^T cell responses played an important role in determining the clinical outcomes of acute self-recovery or long-term viremic persistence following acute HCV infection. Vigorous virus-specific CD4^+ ^T cell responses led to virus clearance in HCV acute-resolved individuals. The failure of effective CD4^+ ^T cell immune responses to clean virus was associated with chronic liver disease and HCV viral persistence. Tregs were defined as a subset of CD4^+^T lymphocytes, highly expressing CD25 (interleukin-2 receptor α-chain) on the cell surface and transcription factor Foxp3 intracellularly and occupied 5% of peripheral CD4^+ ^T lymphocytes [9-11]. Tregs contributed critically to suppression of HCV-specific lymphocyte proliferation, differentiation and cytokine secretion [12-14], but their complicated mechanisms involved in HCV persistence were still obscure.

Another important negative regulating mechanism was the involvement of PD-1/PD-L1 pathway. PD-1 (programmed cell death-1) belongs to the CD28 family and is expressed on activated T, B, and myeloid cells. PD-1 and its ligand PD-L1 deliver inhibitory signals that regulate the balance between effector T cell activation and immune-mediated tissue damage [15,16]. PD-1 knockout C57BL/6 or BALB/c mice were shown to develop lupus-like glomerulonephritis and dilated cardiomyopathy[17,18]and treatment of activated T cells with anti-PD-L1 antibody *in vitro *resulted in reduction of T cell proliferation and IFN-γ secretion[19]. Several studies indicated that PD-1/PD-L1 pathway was critically involved in HCV long-term persistence and was regarded as a potential novel target for restoring function of exhausted HCV-specific T cell responses [20-22].

Since inhibition of the effector T cells by Tregs and the PD-1/PD-L1 pathway were described as mechanisms responsible for balancing of HCV adaptive T cell responses, the association between these two factors should be attractive and informative for the exploration of mechanisms associated with HCV long-term persistence. It was reported that Tregs proliferation and its suppressive function were negatively regulated by PD-1/PD-L1 pathway through a potentially novel mechanism involving the prevention of IL-2 driven STAT-5 phosphorylation during chronic HCV infection [23,24]. However, detailed information with respect to PD-1 expression on different phenotypes (naïve/memory/effector) of Tregs was not well understood.

Not only human CD8^+ ^T cells, but also CD4^+ ^T cells could be divided into four different subsets (naïve, central memory, effector memory and effector) according to expression of maturation marker CD45RA or CD45RO and another co-stimulating molecules (CD62L/CD27/CCR7)[25-29]. CD4^+ ^Treg cells are a specialized subpopulation of CD4^+ ^T cells that act to suppress activation of the immune system and thereby its phenotype and function could be studied through the similar classification principles as used by total CD4^+ ^T cells. Additionally, some groups have already studied the phenotype and function of Treg cells based on expression of CD45RA and/or CD27 molecules[30-33]. In this study, we aimed to investigate the distributional profiles of Tregs subsets according to maturation markers CD45RA and CD27 expression. Association between PD-1 expression on these subsets and development of HCV long-term persistence was also examined. Our findings demonstrated that PD-1 expression on peripheral CD4^+^CD127^lo^CD25^hi ^FoxP3^+ ^Tregs was elevated in HCV chronically infected patients while the extent of elevation was attenuated compared with other CD4^+ ^subsets. Additionally, the dominant proportion of peripheral Tregs carried central memory phenotype in chronic hepatitis C patients compared with nearly equal contribution of naïve and central memory T cell in healthy individuals.

## Materials and methods

### Study population

A total of 19 patients with chronic HCV infection were enrolled in the study. All patients have more than 3-year history of commercial blood donations in early 1990 s. All these patients were positive for HCV antibodies for the past 5 years and previous HCV test data were collected from local CDC and hospital. HCV infection was confirmed by detectable plasma HCV RNA (≥1.48 log_10 _IU/ml) measured by Abbott Real Time™ HCV Amplification Kit (Abbott Molecular Inc. Des Plaines, IL, USA) and positive serum HCV antibodies measured by ARCHITECT Anti-HCV system (Abbott Molecular Inc. Des Plaines, IL, USA) according to the manufacture's instruction. HCV genotype was determined by amplifying and sequencing NS5b gene of HCV genome. CD4^+^/CD8^+ ^T cell counts and liver function associated enzymes were measured based on clinical standard diagnostic criteria by the local Center for Disease Control and Prevention. None of these HCV infected patients received anti-HCV therapy. 17 non-HCV infected individuals were recruited as healthy controls. None of the participants showed evidence of neither concomitant HBV or HIV infection, or other non-viral causes of hepatic damage. The study was approved by institutional review authorities of Peking University Health Science Center and informed consent was obtained from all participants prior to enrollment.

### Flow Cytometry Analysis

Peripheral blood mononuclear cells (PBMC) were isolated from heparinized blood by density gradient centrifugation using Ficoll-Hypaque (Sigma Chemical Co., St Louis, MO, USA). Expression of surface markers on CD4^+ ^T cells were analyzed by multi-parameter flow cytometry on a FACSAria instruction (BD Biosciences, San Jose, CA) compensated with single fluorochromes and driven by FACSDiva software (BD Biosciences, San Jose, CA). The following antibodies were used in this study: anti-CD3-ECD, anti-CD4-APC-Cy7, anti-CD8-PE-Cy7, anti-CD45RA-PE-Cy5, anti-CD27-PE, anti-CD25-APC, anti-CD127-Pacific blue, anti-FoxP3-Alexa700, and anti-PD-1-FITC (BD Pharmingen, San Diego, CA, USA). Data analysis was performed by FlowJo software (Tree Star, Inc., San Carlos, CA).

### Statistical analysis

Characteristics of the study population and the different immune or biochemical parameters were recorded as median and interquartile range. Comparisons between groups were analyzed using non-parametric T tests (Mann-Whitney test) or Wilcoxon match pairs test. Correlations between quantitative parameters were analyzed using Spearman rank correlation coefficient test. All statistical analysis was performed using GraphPad Prism V5.0 (GraphPad Software Inc., San Diego, CA). All P-values were two-tailed, and were considered significant when lower than 0.05.

## Results

### Characteristics of study subjects

Table 1 summarizes the main characteristics of 19 HCV-infected individuals. All data were described as median (IQR1-IQR3). There was no significant difference in age and gender between chronic HCV infection and healthy controls (data not shown).

**Table 1:** Demographics and clinical data of the HCV-infected individuals enrolled in the current study

Characteristics	HCV chronic infection(n = 19)(Median (IQR1-IQR3))
**Age (years)**	**51(40-58)**
**Gender(Male/Female)**	**12/7**
**HCV RNA(log_10_IU/ml)**	**6.14(5.76-6.56)**
**HCV Antibodies (S/CO ratio)**	**14.73(13.64-15.48)**
**HCV Antigens(log_10_fmol/L)**	**3.13(2.84-3.51)**
**Genotype(1b/2a)**	**6/13**
**CD4**^**+ **^**T cell count/μL**	**716(477-950)**
**CD8**^**+ **^**T cell count/μL**	**479(412-686)**
**ALT**^**a **^**level(IU/mL)**	**36(26-50)**
**AST**^**b **^**level(IU/mL)**	**36(32-48)**
**γGT**^**c **^**level(IU/mL)**	**19(17-32)**
**ALP**^**d **^**level(IU/mL)**	**65(56-81)**
**Albumin level(g/L)**	**43.70(41.60-47.20)**
**Total protein level(g/L)**	**76.40(73.14-79.40)**
**Ratio of albumin to globulin**	**1.37(1.18-1.54)**
**Total bilirubin level(μmol/L)**	**13.19(11.00-15.96)**
**Direct bilirubin level (μmol/L)**	**4.41(3.25-5.47)**
**Hemoglobin(g/L)**	**143(131-147)**

^**a**^**ALT, Alanine aminotransferase;**

^**b**^**AST, Aspartate aminotransfearse;**

^**c**^**γ - GT, γ - glutamyltransferase;**

^**d**^**ALP, Alkaline phosphatase**.

### The frequency of different CD4^+^T cell subsets defined by CD127 and CD25, CD45RA and CD27 respectively

**Figure 1:**
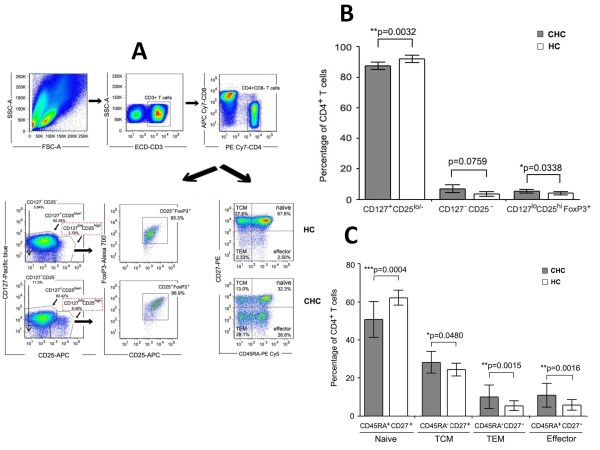
**Increased frequency of Treg cells (CD4^+^CD127^lo^CD25^hi^FoxP3^+^) and effector memory/effector CD4^+ ^T cell subsets in peripheral blood of chronic HCV-infected patients**. PBMCs were stained with T cell phenotyping markers (CD3, CD4, CD8, CD25, CD27, CD45RA, CD127 and FoxP3) and analyzed for T cells subsets by flow cytometric analysis. (**A**): Representative examples of the gating strategy that CD3^+^CD4^+^CD8^- ^T cells were divided into three subsets -CD127^-^CD25^-^, CD127^+^CD25^lo/- ^and CD127^lo^CD25^hi^, based on expression of CD25 and CD127 or into four subsets- naïve, TCM, TEM and effector, based on expression of CD45RA and CD27. Additionally, more than 95% of CD127^lo^CD25^hi ^T cells were further gated as FoxP3^+^. (**B**): Comparison of CD4+ T cell subsets (CD127^-^CD25^-^, CD127^+^CD25^lo/- ^and CD127^lo^CD25^hi ^FoxP3^+^) in chronic HCV infection (*dark grey boxes*) and healthy controls (*open boxes*). (**C**): Comparison of CD4^+ ^T cell subsets (naïve, central memory, effector memory and effector) based on expressions of CD45RA and CD27 in chronic HCV infection (*dark grey boxes*) and healthy controls (*open boxes*). Error bars illustrate s.d. *P < 0.05; **P < 0.01.

Three subsets of CD4^+ ^T cells were defined by the expression of IL-2Rα (CD25) and IL-7Rα (CD127):CD127^+^CD25^lo/-^, CD127^-^CD25^-^, and CD127^lo^CD25^hi ^(Figure 1A). Given that the most definitive markers available to identify Tregs are the co-expression of CD25 and FoxP3, we examined the expression of FoxP3 in the CD127^lo^CD25^hi ^subsets which represented Tregs (Figure 1A). As shown in Figure 1A, more than 95% of CD127^lo^CD25^hi ^subsets were FoxP3 positive cells. The percentage of CD127^lo^CD25^hi^FoxP3^+^Tregs was increased in chronic hepatitis C (CHC) patients compared with health controls (HCs) (*p *= 0.0338) (Figure 1B). On the contrary, decreased expression of CD127^+^CD25^lo/- ^subsets was observed in CHC patients compared with HCs (*p *= 0.0032). No significant difference was found in CD127^-^CD25^- ^subsets distribution (*p *= 0.0759) (Figure 1B). CD4+T cells can be divided into four different subsets depending on expression level of CD45RA and CD27: naïve cells (CD27^+^CD45RA^+^), central memory cells (CD27^+^CD45RA^-^), effector memory cells (CD27^-^CD45RA^-^), and effector cells (CD27^-^CD45RA^+^) (Figure 1A). The statistical results showed that the proportion of naïve CD4^+^T cells were declined in CHC patients compared with HCs (*p *= 0.0004), while the other three subsets (TCM, *p *= 0.0480; TEM, *p *= 0.0015; Effector T, *p *= 0.0016)were all dramatically increased in CHC patients(Figure 1C).

### Expression of PD-1 on different CD4^+^T cell subsets defined by CD27 and CD45RA

**Figure 2:**
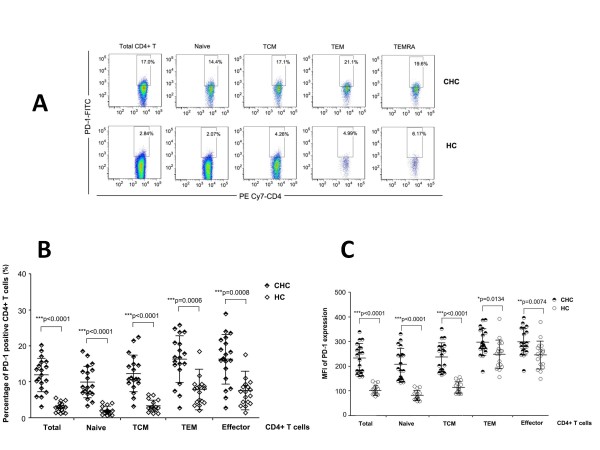
**Percentage of PD-1 positive cells on total CD4^+ ^T cells and its subsets**. (**A**): Representative dot plot analysis showing the expression of PD-1 on total CD4^+ ^T cells and naïve, TCM, TEM, and effector subsets. PD-1 expressions on total CD4^+ ^T cells and all four subsets were significantly higher in chronic HCV-infected patients (*half black symbols*) than in healthy controls (*open symbols*) presented by both percentage (**B**) and MFI (**C**). *P < 0.05; **P < 0.01; ***P < 0.001.

The PD-1 expression on different CD4^+^T cell subsets defined by CD27 and CD45RA was determined by percentage of PD-1 positive cells and mean fluorescent intensity (MFI) of PD-1. PD-1expression was significantly increased in total CD4^+^T cells and all four CD4^+^T cell subsets (naïve/TCM/TEM/effector) in CHC patients compared with HCs (Figure 2A, B, C). Of note, expression of PD-1 was higher on TEM/effector subsets than naive/TCM subsets in both CHC patients and HCs (Figure 2B and 2C).

### Expression of PD-1 on CD4^+^CD127^lo^CD25^hi^FoxP3^+ ^Tregs

**Figure 3:**
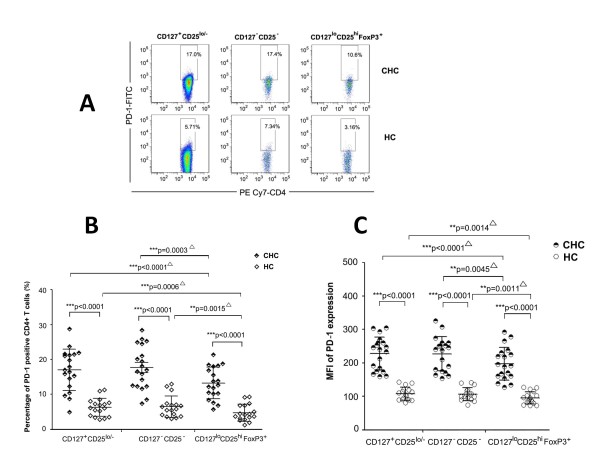
**Percentage of PD-1 positive cells on CD127^-^CD25^-^, CD127^+^CD25^lo/- ^and CD127^lo^CD25^hi ^FoxP3^+ ^CD4+ T cell subsets**. (**A**): Representative dot plot analysis showing the expression of PD-1 on these three subsets. PD-1 expressions on these T cell subsets were significantly higher in chronic HCV-infected patients (half black symbols) than in healthy controls (*open symbols*) presented by both of percentage (**B**) and MFI (**C**). PD-1 expression on Treg cells (CD127^lo^CD25^hi ^FoxP3^+^) was lower than on paired CD127^-^CD25^- ^and CD127^+^CD25^lo/- ^subsets. ^▵^indicated that Wilcoxon match pairs test. *P < 0.05; **P < 0.01; ***P < 0.001.

We next examined and compared the expression profile of PD-1 on different CD4^+^T cell subsets defined by CD127 and CD25. PD-1 expression was remarkably higher on all three CD4+T cell subsets (CD127^+^CD25^lo/-^, CD127^-^CD25^-^, and CD127^lo^CD25^hi^FoxP3^+^) in CHC patients compared to HCs (*p *< 0.0001) (Figure 3A, B and 3C). In addition, there was a significant decrease of PD-1 expression on CD127^lo^CD25^hi^FoxP3^+ ^Tregs compared with CD127^+^CD25^lo/- ^(*p *< 0.0001 on percentage and MFI) or CD127^-^CD25^-^(p = 0.0003 on percentage and p = 0.0045 on MFI) T cell subsets in CHC patients (Figure 3B and 3C). The similar trend was found in CHC patients (Figure 3B and 3C).

### The dominance of the central memory phenotype in CD4^+^CD127^lo^CD25^hi^FoxP3^+ ^Tregs in CHC patients

**Figure 4:**
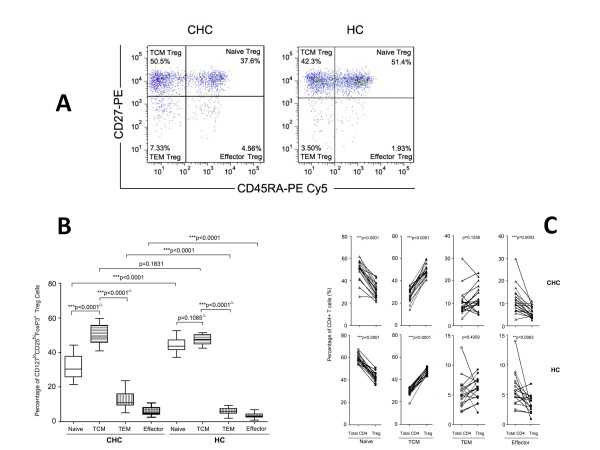
**The dominant proportion of the central memory phenotype was found in CD127^lo^CD25^hi^FoxP3^+ ^Tregs in CHC patients**. (**A**) Representative dot plot analysis showing the phenotypic distribution of CD127^lo^CD25^hi^FoxP3^+ ^Tregs depending on expression of CD27 and CD45RA. (**B**) Frequency of Naïve -Tregs (□) was dramatically reduced in CHC patients compared with HCs while TEM (▥)/Effector (▦) -Tregs showed a higher levels in CHC compared with in HCs. (**C**) The percentage of naïve and effector subset decreased while the percentage of TCM subset increased in Tregs compared with in their total CD4^+ ^T cell counterparts. *P < 0.05; **P < 0.01; ***P < 0.001.

CD127^lo^CD25^hi^FoxP3^+ ^Tregs were divided into Naïve-Tregs, TCM-Tregs, TEM-Tregs, Effector-Tregs depending on their expressions of CD27 and CD45RA (Figure 4A). As shown in Figure 4B, the frequency of Naïve-Tregs was dramatically reduced in CHC patients compared with HC individuals (*p *< 0.0001), while expression of TEM/Effector-Tregs was dramatically enhanced in CHC patients compared with HCs (*p *< 0.0001). No statistical difference was observed regarding to the proportion of TCM-Tregs in total CD4^+^Tregs between CHC patients and HCs (Figure 4B). It should be pointed out that the highest proportion (nearly 50%) of Tregs carried central memory phenotype in CHC patients due to a decreased proportion of naïve-Tregs (Figure 4B).

In addition, the distributional pattern of different CD4^+^T cell subsets was evaluated and compared in total CD4^+^T cells and Tregs. The frequency of naïve cells displayed significantly declined trends from total CD4^+^T cells to Tregs (*p *< 0.0001) in both CHC and HCs. In contrast, a significantly increased frequency of TCM phenotype was found in Tregs compared with total CD4^+^T cells (*p *< 0.0001) (Figure 4C). The proportion of terminal differentiated effector cells was downregulated in Tregs (*p *= 0.0003 in CHC and *p *= 0.0063 in HCs) while no difference in TEM frequency was found between total CD4^+^T cells and Tregs (Figure 4C). Therefore, this results indicated that central memory CD4^+^T cells was the dominant subset in Tregs, instead of naïve CD4+T cells which contributed to the majority of subset in total CD4^+^T cells.

### Expression of PD-1 on different CD4^+^CD127^lo^CD25^hi ^FoxP3^+ ^Tregs subsets defined by CD27 and CD45RA

**Figure 5:**
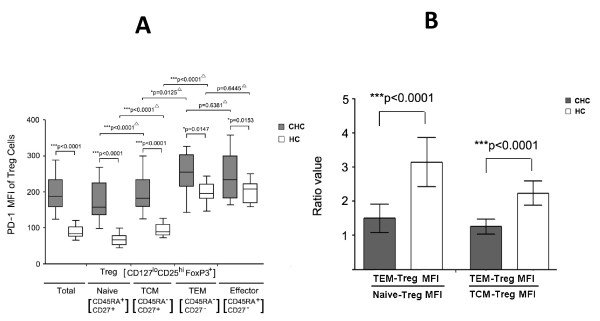
(**A**) Increased MFI of TEM/effector Treg cells compared with naïve/TCM Treg cells in CHC patients and HC controls; (**B**) Ratios of TEM-Tregs to naive-Tregs and TEM-Tregs to TCM-Tregs regarding to PD-1 MFI were significantly lower in CHC patients than in HCs. ^▵^indicated that Wilcoxon match pairs test. *P < 0.05; **P < 0.01; ***P < 0.001.

PD-1 expression on different Tregs subsets was studied. PD-1 expression was significantly higher on total Tregs (*p *< 0.0001) and all four Tregs subsets (Naïve, *p *< 0.0001; TCM, *p *< 0.0001; TEM, *p *< 0.0147; Effector, *p *< 0.0153) in CHC patients compared with HCs. Besides, PD-1 expression was higher in TEM/effector-Tregs compared with naïve/TCM-Tregs. TCM Tregs showed lower PD-1 expression than TEM subsets (CHC, *p *= 0.0125; HC, *p *< 0.0001) while higher than naïve subsets in both CHC patients and HCs (CHC, *p *< 0.0001; HC, *p *< 0.0001) (Figure 5A).

Since PD-1 expression was enhanced on all CD4^+^T cell subsets on both chronic HCV infected patients and healthy controls, it is necessary and informative to compare the ratio of TEM-Tregs to naive-Tregs and of TEM-Tregs to TCM-Tregs regarding to PD-1 MFI between CHC patients and HCs. The results shown in Figure 5B indicated both TEM-Tregs/naïve-Tregs ratio and TEM-Tregs/TCM-Tregs ratio were significantly lower (*p *< 0.0001) in CHC patients than health controls. Unfortunately, no correlation was found between these ratios and peripheral HCV RNA level (data not shown).

## Discussion

Both CD4^+^CD127^lo^CD25^hi ^and CD4^+^FoxP3^+^were classic phenotypes of regulatory CD4^+ ^T cells, which has been described by accumulating literatures [34-36]. In the present study, we employed CD4^+^CD127^lo^CD25^hi^FoxP3^+ ^cells as circulating natural Tregs to investigate distributional characteristics of naïve, memory and effector subsets of Tregs. PD-1 expression on Tregs and its subsets was also studied in chronic HCV infected patients and healthy controls. Previous studies demonstrated human Tregs could be phenotypically and functionally divided into resting Tregs (rTregs) and activated Tregs (aTregs) according to the expression of surface CD45RA. Both rTregs and aTregs were shown to suppress proliferation of CD25^- ^CD4^+ ^responder T cells *in vitro *and CD45RA^- ^FoxP3^low/- ^subsets were non-Treg cells since they had no suppressive function [30]. Herein, our study investigated the classification of Tregs based on the expression of CD45RA and CD27 (a TNFR family member) since total CD4^+ ^T cells could be subdivided into four different subsets (naïve/central memory/effector memory/effector) according to the expression of CD45RA and CD27. Our results (Figure 4) indicated that the proportion of Tregs in peripheral bulk CD4^+ ^T cells was enhanced in CHC patients. The phenotypic characteristics of Tregs were switched from nearly equal contribution of naïve and central memory T cell of peripheral Tregs in HCs to central memory T cell contributing to be the dominant one in CHC patients, suggesting that peripheral Tregs of CHC patients were changed quantitatively and functionally.

PD-1/PD-L1 pathway was proved to play a critical role in maintaining the balance between protective T cell responses and immunopathology induced by hyper-activated effector T cells [15,16,20,37-40]. It has been reported that CD4^+ ^T-cell responses, including virus-specific IFN-γ production, were severely suppressed in chronically HCV-infected subjects through PD-1/PD-L1 pathway [20]. Though PD-1 was demonstrated to be expressed on Tregs and negatively regulated CD4^+^CD25^+^Foxp3^+ ^Tregs function by preventing STAT-5 phosphorylation in CHC patients [23], it was still far from well understanding the molecular mechanisms of PD-1/PD-L1 pathway involving in inhibition of CD4^+ ^T cell function, including Foxp3^- ^effector T cells, Foxp3^- ^memory T cells and Tregs. Our data showed that PD-1 expression on total Tregs as well as its subsets was significantly higher in HCV infected patients than healthy controls (Figure 5A). This results were not consistent with the hypothesis that chronic HCV infection was associated with higher inhibitory effect of Tregs since proliferation of Tregs was inhibited by PD-1 and subsequent IL-2-driven STAT-5 phosphorylation. With further investigation, we found (Figure 5B) that the TEM-Tregs/naive-Tregs ratio and TEM-Tregs/TCM-Tregs ratio of PD-1 MFI were significantly lower in CHC patients than healthy controls, indicating that PD-1 expression on TEM-Tregs or effector-Tregs were inhibited in relative to its correspondent naïve- or TCM-Tregs in CHC patients compared with healthy controls. Theoretically, the relatively reduced PD-1 expression on TEM-Tregs and effector-Tregs could enhance the inhibitory capacity of activated Tregs on hyper-activated effector T cells, attributing to impaired T cell immune response and subsequently inducing long-term persistence of HCV infection.

Taken together, our findings provided insight into the negative regulatory mechanism of Tregs and PD-1 on CD4^+^T cell responses during chronic HCV infection. We demonstrated that most Tregs displayed a central memory phenotype and PD-1 expression had a trend of relatively lower upregulated expression on effector Tregs and effector memory Tregs in relative to their naïve- or TCM-Tregs counterparts in chronic HCV infected patients compared with healthy individuals. The cross talk between Treg cells and PD-1 induced inhibition in chronic HCV infection and its implication in dysfunction or impairment of CD4^+ ^T cell responses should be interesting and suggestive for further exploration of persistent HCV infection associated immune pathogenesis.

## Authors' contributions

TS, HL and FL design the study. TS and JZ performed the statistical analysis and interpretation of the data. TS and HL drafted the manuscript. TS, JZ, CX, TZ, and QX collected samples and performed benchwork. All suthors read and approved the final manuscript.

## Competing interests

The authors declare that they have no competing interests.
